# Novel Approach to Bile Duct Damage in Primary Biliary Cirrhosis: Participation of Cellular Senescence and Autophagy

**DOI:** 10.1155/2012/452143

**Published:** 2011-07-07

**Authors:** Motoko Sasaki, Yasuni Nakanuma

**Affiliations:** Department of Human Pathology, Kanazawa University Graduate School of Medicine, Kanazawa 920-8640, Japan

## Abstract

Primary biliary cirrhosis (PBC) is characterized by antimitochondrial autoantibodies (AMAs) in patients' sera and histologically by chronic nonsuppurative destructive cholangitis in small bile ducts, eventually followed by extensive bile duct loss and biliary cirrhosis. The autoimmune-mediated pathogenesis of bile duct lesions, including the significance of AMAs, triggers of the autoimmune process, and so on remain unclear. We have reported that cellular senescence in biliary epithelial cells (BECs) may be involved in bile duct lesions and that autophagy may precede the process of biliary epithelial senescence in PBC. Interestingly, BECs in damaged bile ducts show characteristicsof cellular senescence and autophagy in PBC. A suspected causative factor of biliary epithelial senescence is oxidative stress. Furthermore, senescent BECs may modulate the microenvironment around bile ducts by expressing various chemokines and cytokines called senescence-associated secretory phenotypes and contribute to the pathogenesis in PBC.

## 1. Introduction

Primary biliary cirrhosis (PBC) is a chronic, progressive cholestatic liver disease that affects usually middle-aged women and occasionally leads to liver failure and liver transplantation [1–5]. Autoimmune pathogenesis is suggested in PBC [1–4], because PBC is serologically characterized by a high titer of serum antimitochondrial autoantibodies (AMAs) and by an increased level of immunoglobulin M (IgM). PBC-specific antinuclear antibodies (ANAs), such as anti-gp210 are also detected in some patients [1, 2, 6–9]. AMAs are present in about 95% of patients with PBC, with disease specificity close to 100%. An inner lipoyl domain of the E2-component of pyruvate dehydrogenase (PDC-E2) and other 2-oxo-acid dehydrogenases is a major epitope for both B-cell and CD4 and CD8 T-cell response [9–12]. PBC is characterized histologically by the cholangitis of small bile ducts (chronic nonsuppurative destructive cholangitis; CNSDC), eventually followed by the extensive loss of small bile ducts and biliary cirrhosis [2, 3, 13]. Therefore, a major target of autoimmune-mediated injury has been thought to be biliary epithelial cells (BECs) in PBC.

There has been considerable progress in elucidating the immunopathological features [9–12], genetic factors [14–17], and environmental factors such as infectious agents and xenobiotics [5, 18–20] in the pathogenesis of PBC. The most accepted hypothesis states that PBC results from a combination of multiple genetic factors (susceptible genetic background) and superimposed environmental triggers. In this scenario, adaptive, both humoral and cellular (CD4 and CD8 T cells), and innate immunity have been proposed as coplayers in immune-mediated liver damage; however, the etiology and pathogenesis of PBC remain unclear. In particular, the significance of AMAs and autoantigen-specific T-cell response in the pathogenesis of bile duct lesions remains unknown. One hypothesis for a BEC-specific autoimmune reaction is a unique property of apoptosis in BECs, in which there is exposure of autoantigen to the effectors of the immune system [4, 21–23].

We have recently reported that cellular senescence and autophagy may be involved in bile duct lesions in PBC [24–28]. These two cellular processes may be related to autoimmune mechanism such as AMAs and the autoantigen-specific T cell and play a role to cause autoimmune-mediated bile duct lesions in PBC. Recent studies have disclosed that autophagy plays an important role in innate immune responses and possibly autoimmunity [29–31]. Furthermore, it is plausible that senescent BECs modulate microenvironment around bile duct by expressing senescence-associated secretory phenotypes (SASPs) including various chemokines and contribute to the pathogenesis of bile duct lesions in PBC [32]. In this paper, we will focus on cellular senescence and autophagy in BECs in PBC and their possible involvement in the progression of diseases.

## 2. Cellular Senescence in the Damaged Small Bile Ducts in PBC

### 2.1. What Is Cellular Senescence?

**Figure 1:**
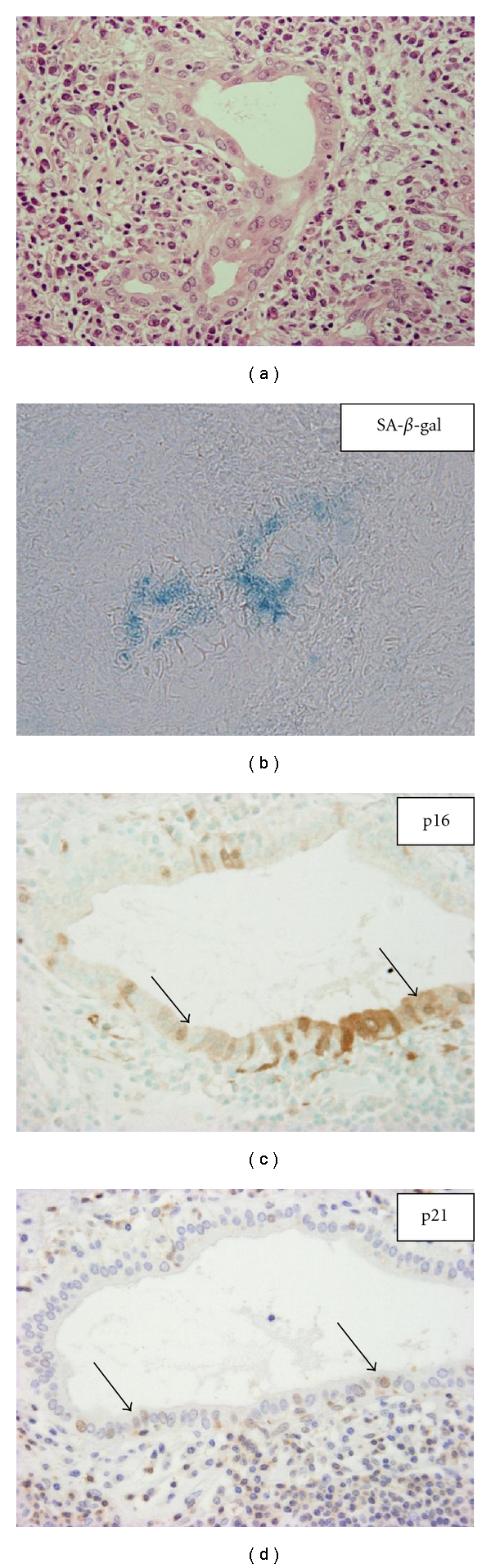
Biliary epithelial senescence in PBC. BECs in small bile ducts involved in chronic nonsuppurative destructive cholangitis (CNSDC) show histological features of senescence, such as cytoplasmic eosinophilia, cellular and nuclear enlargement, and uneven nuclear spacing (a). SA-*β*-gal activity is detected in BECs in PBC (b). Senescent markers, p21^WAF1/Cip1^ and p16^INK4a^, were expressed in BECs in damaged small bile ducts in PBC (c) and (d). Immunostaining for p21^WAF1/Cip1^ and p16^INK4a^. Original magnification: ×400.

Cellular senescence is defined as a condition in which a cell no longer has the ability to proliferate. Senescent cells remain metabolically active, even though they are irreversibly arrested at the G1 phase of the cell cycle and do not respond to various external stimuli. Cellular senescence can be triggered by a number of cellular stresses including telomere dysfunction. Other causes include oxidative stress, nontelomeric DNA damage, epigenetic derepression of the INK4a/ARF locus, and oncogenic activation [33]. Several features, such as increased activity of senescence-associated *β*-galactosidase (SA-*β*-gal) (Figure 1), shortened telomeres, increased expression of p16^INK4^ and p21^WAF1/CIP^, and histological changes (Figure 1), are known to characterize cellular senescence [34–36]. Cellular senescence is a potent tumor suppression mechanism as well as apoptosis [33, 37]. Senescent cells are also seen in aged or damaged tissues, and they may decline tissue regeneration capacity with age [33]. Cellular senescence may play a role in limiting wound-healing responses following tissue damage [38]. Recent studies have disclosed that cellular senescence is involved in the pathophysiology of various chronic liver diseases, including chronic viral hepatitis and hepatocarcinogenesis [24, 26, 27, 38–44].

### 2.2. Bile Duct Lesion in PBC

“Chronic nonsuppurative destructive cholangitis (CNSDC)” is a characteristic bile duct lesion in PBC (Figure 1(a)) [2, 3, 13, 45]. Bile duct damage in early PBC mainly affects the septal and larger interlobular bile ducts, while the smaller interlobular ducts remain intact until later. The BECs in the affected bile ducts show irregular shape and arrangement with infiltration of mononuclear cells. The presence of epithelioid granuloma around the affected bile duct is also a feature of PBC. Bile duct loss eventually progresses and chronic cholestasis developes gradually. Hepatitis activity of varying degrees is frequently imposed on the liver at the same time. We proposed a new histological staging and grading system of PBC for comprehensive analysis of the histological progression of PBC (staging) toward extensive bile duct loss, chronic cholestasis and cirrhosis, and also the immune-mediated necroinflammatory activity of small bile ducts and hepatocytes [46].

### 2.3. Biliary Epithelial Senescence in Damaged Small Bile Ducts in PBC

BECs in damaged small bile ducts in PBC show senescent features, such as the expression of SA-*β*-gal and the increased expression of p16^INK4a^ and p21^WAF1/Cip1^ (Figure 1) [24–27]. Furthermore, a significant decrease in telomere length was observed in BECs in the damaged small bile ducts and bile ductules in PBC compared with normal-looking bile ducts and bile ductules in PBC, chronic viral hepatitis, and normal livers, when examined using quantitative fluorescence *in situ* hybridization [25]. *γ*H2AX DNA damage foci were detected in BECs in damaged small bile ducts and bile ductules in PBC but were absent in BECs in control livers. The expression of p16^INK4a^ and p21^WAF1/Cip1^ increased corresponding to telomere shortening and *γ*H2AX DNA damage foci in the damaged small bile ducts in PBC [25]. Taken together, telomere shortening and the accumulation of DNA damage coinciding with increased expressions of p16^INK4a^ and p21^WAF1/Cip1^ in the damaged bile ducts characterize biliary cellular senescence and may play a role in subsequent progressive bile duct loss in PBC [24–27]. Interestingly, chronic liver allograft rejection, which is characterized by bile duct loss similar to PBC, also shows similar biliary epithelial senescence [24, 40].

### 2.4. How Does Cellular Senescence Result in Bile Duct Loss?

The exact mechanism how cellular senescence of BECs cause bile duct loss in PBC is not clear. Cellular senescence is supposed to impair tissue integrity and cause persistent inflammation [47]. After cellular senescence occurs in injured BECs, these senescent cells are thought to remain *in situ *and not to be replaced by normal cells, although nonsenescent BECs proliferate in response to injury [48]. Therefore, it is plausible that the senescent BECs are prone to further injuries, accentuating inflammation by SASP, which is likely to be followed by bile duct loss in PBC. The fate of senescent BECs remains to be clarified: whether senescent BECs are removed by necrosis, apoptosis, or anoikis. Another possibility is that bile duct loss may be due to impaired function of hepatic stem/progenitor cells in PBC. Cellular senescence is also seen in bile ductular cells in a ductular reaction (DR), which is thought to harbor hepatic stem/progenitor cells in PBC [24, 25]. The impaired proliferation of hepatic stem/progenitor cells may fail to replace the damaged BECs in small bile ducts, subsequently cause bile duct loss.

### 2.5. Oxidative Stress Is a Potential Factor Inducing Cellular Senescence

Cellular senescence can be triggered by a number of cellular stresses, including telomere dysfunction, oxidative stress, nontelomeric DNA damage, epigenetic derepression of the INK4a/ARF locus, and oncogenic stress [33, 39]. The possible association of oxidative stress is suggested to be involved in the pathogenesis of cellular senescence in PBC [24, 26, 27]. For example, p21^WAF1/Cip1^, activated/phosphorylated ATM, and an oxidative stress marker, 8-OHdG, were frequently and extensively coexpressed in the nuclei of CNSDC in PBC, and their expressions were correlated [26]. Cell culture study suggests that oxidative stress and proinflammatory cytokines, such as IFN-*β*, IFN-*γ*, and TNF-*α*, which induce ROS generation, activate the ATM/p53/p21^WAF1/Cip1^ pathway, followed by biliary epithelial senescence [49]. The expression of polycomb group protein Bmi1 is significantly decreased in damaged bile ducts in PBC, coordinating with the increased expression of p16^INK4a^ [27]. The decreased expression of Bmi1 is induced by oxidative stress, followed by the increased expression of p16^INK4a^ in cultured BECs [27]. Since an antioxidant, N-acetylcysteine can inhibit cellular senescence induced by oxidative stress and proinflammatory cytokines [49], antioxidants may have therapeutic implications in PBC.

## 3. Cellular Senescence in Ductular Reaction (DR) in PBC

**Figure 2:**
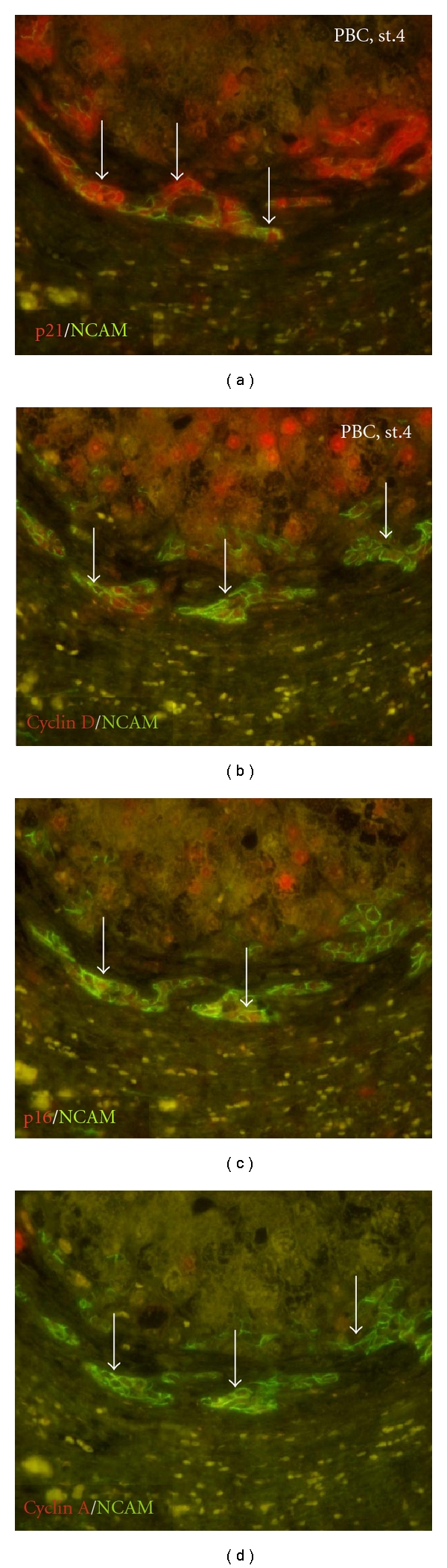
Double immunostaining for senescence markers (p16^INK4a^ or p21^WAF1/Cip1^) and cell cycle markers (G1-phase, cyclin D; S-phase, cyclin A) (red) and NCAM (green) in PBC, stage 4. (a), (c) The expression of senescent markers p16^INK4a^ and p21^WAF1/Cip1^ is seen in NCAM-positive ductular cells (arrows) in PBC, stage 4. (b), (d) Most NCAM-positive ductular cells (arrows) express cyclin D, whereas there is no cyclin A expression in DRs in PBC, stage 4. Original magnification ×400.

DR is a reactive lesion at the portal tract interface composed of increased bile ductules with an accompanying complex of stromal and inflammatory cells [50]. DR is thought to harbor hepatic stem/progenitor cells [50]. We investigated the pathological significance of DR in chronic liver diseases, including PBC, with respect to cellular senescence [24, 25, 51]. The expression of senescence-associated markers (p16^INK4a^ and p21^WAF1/Cip1^) was frequently expressed in ductular cells in the advanced stage of chronic liver diseases, especially in PBC. Double immunostaining disclosed that neural cell adhesion molecules (NCAM) were frequently coexpressed in ductular cells showing senescence-associated markers (p16^INK4a^ and p21^WAF1/Cip1^) and cell cycle G1-phase marker (cyclin D) (Figure 2) [51]. These findings suggest that DR is heterogeneous in cell kinetics and the expression of NCAM and that some ductular cells in DR in chronic liver diseases were at G1 arrest and undergoing cellular senescence. Such senescent cells may be involved in the progression of fibrosis of these diseases, particularly in PBC [51]. This study raises the possibility that NCAM can be used as a cellular senescent marker developing in DRs. Furthermore, our recent study revealed that CCL2 expressed by senescent BECs can induce the cell migration of hepatic stellate cells (HSCs), which may play a role in the periportal fibrosis in chronic advanced liver diseases [52].

## 4. Autophagy in Damaged Small Bile Ducts in PBC

### 4.1. What Is Autophagy?

Autophagy, or cellular self-digestion, is a cellular pathway that results from various cellular stresses, such as nutrient starvation, anoxia, and activation of the endoplasmic reticulum stress pathway [53, 54]. Three types of autophagy, macroautophagy, microautophagy, and chaperone-mediated autophagy, have been classified, and macroautophagy is the major type [53–55]. It is becoming evident that macroautophagy (hereafter referred to as autophagy) is important for development, differentiation, survival, homeostasis, and also many pathological processes. Autophagy occurs physiologically at low basal levels in cells to perform homeostatic functions such as protein and organelle turnover. It is rapidly upregulated through an inhibition of mammalian target of rapamycin (mTOR) when cells need to generate intracellular nutrients and energy, for example, in starvation [53–55]. Microtubule-associated protein-light chain 3*β* (LC3), a homologue of autophagy-related protein 8 (Apg8p), which is essential for autophagy and associated with autophagosome membranes after processing, is a widely used marker of autophagy [56, 57].

### 4.2. Cellular Senescence, Apoptosis, and Autophagy

An appropriate cellular stress response is critical for maintaining tissue integrity and function and for preventing diseases [58]. Cellular senescence, apoptosis, and autophagy are cellular responses to stress, correlating with each other [58]. Cellular stresses cause adaptation, repair, autophagy, apoptosis, or cellular senescence in cells [58]. These cell fate decisions are critical to dealing with the emergence of damaged and potentially dangerous cells that can cause cancer. Interestingly, a recent study disclosed that autophagy is induced during and facilitates the process of senescence [56]. Cellular senescence can be a failsafe program against a variety of cellular insults, as well as apoptosis. Cellular senescence is a typical delayed stress response involving multiple effector mechanism, in contrast, cytotoxic signals converge to a common mechanism in apoptosis. With the onset of cellular senescence cells can remain viable within tissues for long periods; resistance to apoptosis is a characteristics of senescent cells [41, 59].

### 4.3. Biliary Epithelial Autophagy in PBC

**Figure 3:**
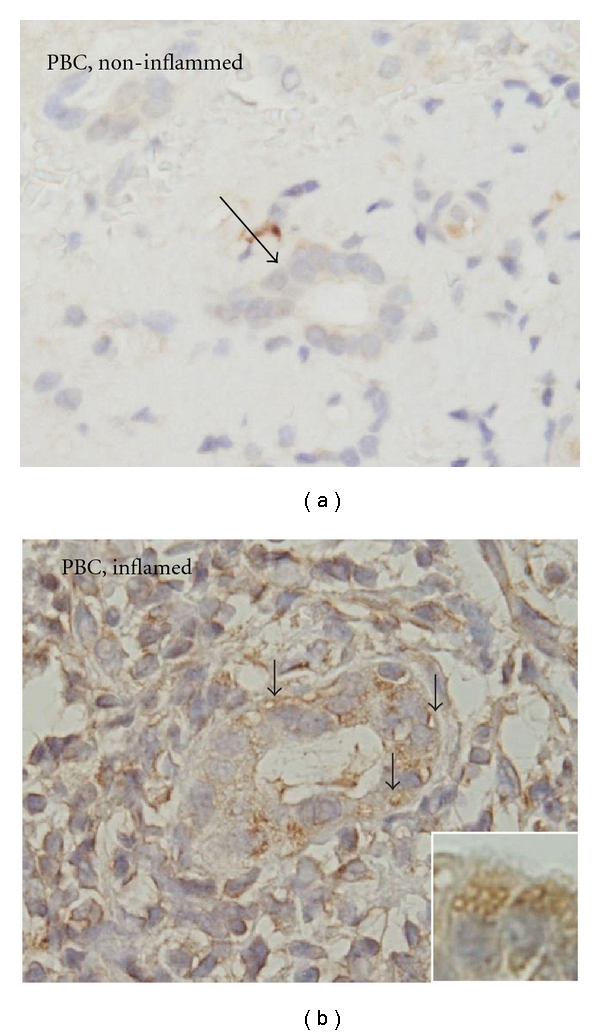
Biliary epithelial autophagy in PBC. (a) The expression of autophagy marker LC3 was not observed in BECs in noninflammed bile ducts (arrow) in PBC. (b) The expression of autophagy marker LC3 was detected in intracytoplasmic vesicles (arrows) in BECs involved in inflamed and damaged small bile ducts in PBC. Immunostaining for LC3. Original magnification, ×400 (inset, ×1000).

We have reported the upregulated autophagy in the damaged small bile ducts along with cellular senescence in PBC [28] (Figure 3). LC3, a commonly used marker of autophagy, was characteristically expressed in cytoplasmic vesicles in bile duct lesions in PBC [28]. Autophagic marker LC3 was coexpressed with senescent markers p21^WAF1/Cip1^ and p16^INK4a^ in damaged bile ducts in PBC [28]. The inhibition of autophagy reduced stress-induced cellular senescence in cultured cells with stress [28]. This finding is in consistent with a recent study in which the involvement of autophagy is reported in the process of senescence [56]. Taken together, biliary epithelial autophagy may mediate the process of biliary epithelial senescence in bile duct lesions in PBC and it may be involved in the pathogenesis of bile duct lesions in PBC.

### 4.4. Autophagy and Autoimmune-Mediated Processes in PBC

An unsolved problem is how autophagy and cellular senescence are involved in the autoimmune-mediated processes such as AMA and other PBC-related autoantigens in PBC. Regarding apoptosis, it has been reported that BECs manifest unique features during apoptosis and that the combination of AMA and BECs apoptotic bodies (apotopes) could activate innate immune response with involvement of some inflammatory cytokines [21]. This study provides a mechanism for the biliary specificity of PBC and the involvement of AMA in autoimmune pathogenesis [21]. Recent studies reveal a crucial role for the autophagy pathway and proteins in immunity and inflammation [29–31]. The autophagy pathway and autophagy proteins may function as a central fulcrum that balances the beneficial and harmful effects of the host response to infection and other immunological stimuli [31]. Autophagy proteins function in adaptive immunity, including in the development and homeostasis of the immune system and in antigen presentation [31]. Furthermore, autophagy proteins play a role in both the activation and inactivation of innate immune signaling [30, 31]. On the contrary, it is demonstrated that autophagy is regulated by immune-signaling molecules, such as toll-like receptors (TLRs), IFN-*γ*, and NF-*κ*B [30, 31].

The dysfunctional autophagy related to the regulation of immunity may contribute also to chronic inflammatory diseases and probably autoimmune diseases. A well-characterized link is between mutations in autophagy regulators and Crohn's disease, a chronic inflammatory bowel disease, in which autophagy proteins, ATG16L1, NOD2, and IRGM are reported as susceptibility genes [60]. Abnormal autophagy/autophagy protein may also result in inflammatory autoimmune disease, although not yet proven. Autophagy-related processing of self-proteins provides a source of immunostimulatory molecules and autoantigens, that is, by MHC-class II presentation of cytosolic antigens and control of T-cell homeostasis [61–63]. It is of interest that genomewide association studies (GWAS) have linked several single nucleotide polymorphisms (SNPs) in ATG5, an autophagy protein, to systemic lupus erythematosus (SLE) susceptibility [64, 65]. SLE is a representative multisystem autoimmune disease characterized by an enormous array of autoantibodies such as ANAs and autoimmune responses against self-antigens generated from dying cells. To date, it is unclear how such SNPs affect the expression level and function of ATG5. Interestingly, in mice, the lack of ATG5-dependent negative thymic selection generates autoimmunity and multiorgan inflammation [66]. The autoimmunity and inflammation associated with SLE may be caused by loss of other ATG5-dependent effects, such as regulation of IFN and proinflammatory cytokine secretion, clearance of dying cells [67], and dendritic cell antigen presentation [68]. Taken together, a link between SLE pathogenesis and ATG5 mutation or mutation of other autophagy genes is plausible, although not yet proven. 

Similar to SLE, it is possible that a dysfunctional autophagic process of BECs may play a role in autoimmune pathogenesis, for example, the immune tolerance breakdown of autoantigens, in PBC, although this is only speculative at this moment. Recent genetic studies of PBC including GWAS identified, in a reproducible fashion, genetic associations between PBC and human leukocyte antigen as well as polymorphisms in the genes encoding IL-12 *α*-chain and IL-12 receptor *β*-chain [14, 15]. GWAS also identified interferon regulatory factor 5 (IRF5)-transportin 3 (TNPO3), 17q12-21, MMEL1, and SPIB as new PBC susceptibility loci [14, 15]. These immune-related genes may be associated with dysfunction autophagy in PBC, although there have been no identified autophagy proteins such as ATG5 and ATG16L1 as PBC susceptibility genes. In fact, IRF5 plays a key role in the innate immunity response as part of the TLR signaling pathway and mediates apoptosis induced by tumor necrosis factor-related apoptosis-inducing ligand [69, 70]. Interestingly, IRF5 loci have been reported as associated loci with several autoimmune diseases including SLE and Sjogren's syndrome [71, 72]. Therefore, IRF5 might be related to dysfunctional autophagy in PBC, although not yet reported.

## 5. Senescence-Associated Secretory Phenotypes (SASPs) in PBC

### 5.1. What Are SASPs?

An increasing body of work described the change in the cellular secretosome in senescent cells. Senescent cells play an important role in modulating the microenvironment by secreting biological active molecules, senescence-associated secretory phenotypes (SASPs). SASPs include diverse proinflammatory factors such as cytokines (IL-6, IL-1 and so on) and chemokines (CXCL8/IL-8, CCL2/monocyte chemotactic protein-1 (MCP)-1) and so on), growth factors and profibrogenic factors [73–77]. Previous studies have shown that BECs express a number of profibrogenic proinflammatory and chemotactic factors (e.g., IL-1, IL-6, CXCL8/IL-8, and CCL2/MCP-1) [78–81]. These factors can attract and activate inflammatory cells and also stellate cell lineage in humans with biliary disorders and in animal models of biliary fibrosis. Taken together, these cytokines and chemokines previously reported in PBC may belong to SASPs [73–77]. 

### 5.2. SASPs May Play a Role in the Pathogenesis of PBC

**Figure 4:**
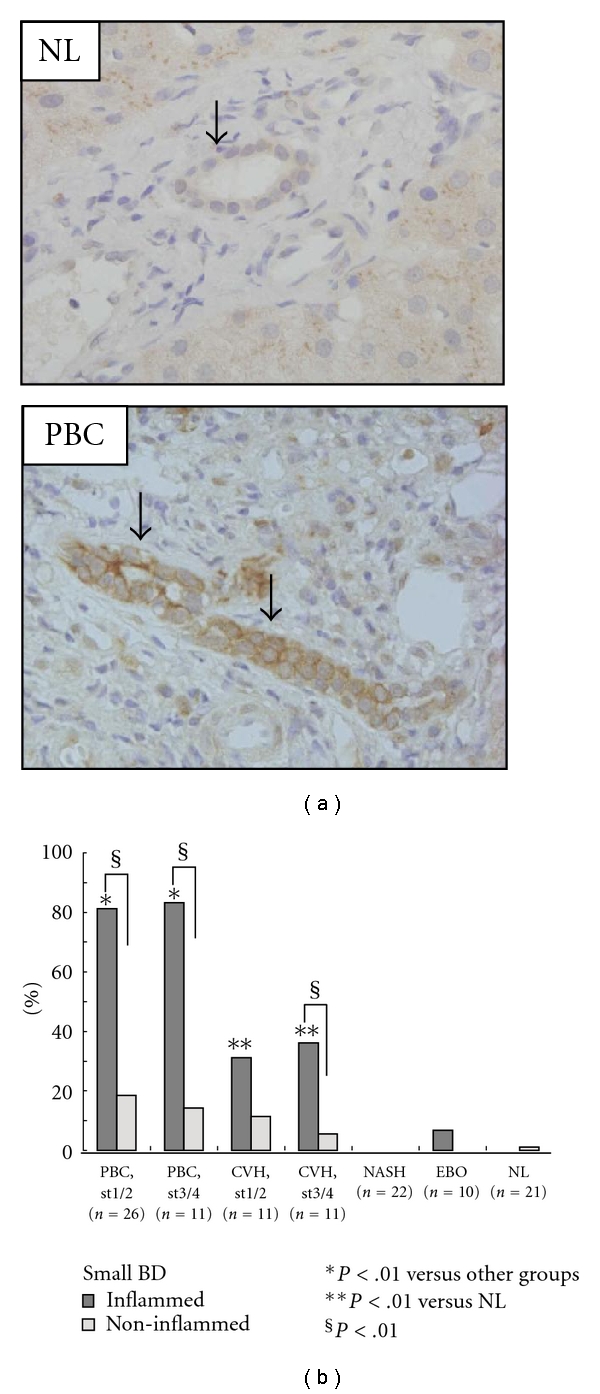
Increased expression of CCL2 in inflamed and damaged bile ducts in PBC. (a) The expression of CCL2 was absent or faint in biliary epithelial cells (BECs) in the small bile duct (arrow) in normal liver (top). CCL2 was extensively expressed in the membrane and cytoplasm of damaged and senescent BECs (arrows) in the early stage of PBC (bottom). Immunostaining for CCL2. Original magnification, ×400. (b) The expression of CCL2 was significantly more frequent and intense in inflamed small bile ducts in PBC, when compared with noninflamed small bile ducts in PBC and small bile ducts in control livers (*P* < .01). CVH: chronic viral hepatitis; NASH: nonalcoholic steatohepatitis; EBO: extrahepatic biliary obstruction; NL: normal liver.

**Figure 5:**
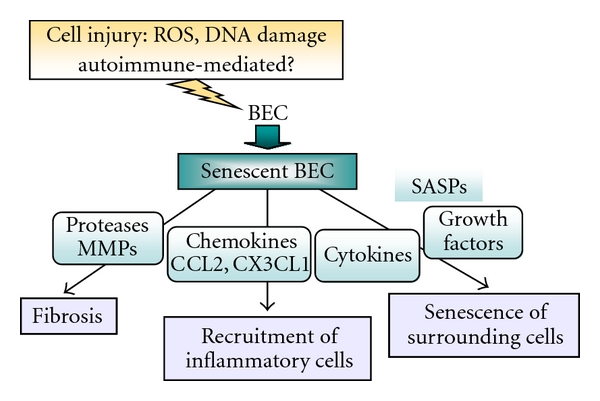
Possible regulation of microenvironment by senescent BECs expressing SASPs in PBC. Senescent BECs may function in modulation of the inflammatory microenvironment by recruiting monocytes and possibly other inflammatory cells by secreting chemokines and cytokines as SASPs. Senescent BECs may also participate in the induction of senescence in surrounding cells and progression of fibrosis via SASPs.

The upregulation of several cytokines and chemokines in damaged bile ducts in PBC has been reported [79, 80, 82], and these factors may represent SASPs, as described above [73–77]. We have recently reported that the involvement of senescent BECs in modulation of the inflammatory microenvironment around affected small bile ducts in PBC (Figure 4) [32]. In this study, we have shown that the expression of CCL2 and CX3CL1 was significantly higher in BECs in inflamed and damaged small bile ducts in PBC, than in noninflamed bile ducts and control livers (Figure 4). The expression of CCL2 and CX3CL1 was colocalized with the expression of senescent markers in damaged bile ducts in PBC [32]. In culture study, senescent BECs induced by cellular stresses expressed a significantly higher level of chemokines. Furthermore, senescent BECs significantly accelerated the migration of RAW264.7 cells, and neutralizing antibodies against CCL2 and CX3CL1 blocked in part the migration induced by senescent BECs [32]. These findings suggest that senescent BECs may play an important role in the pathogenesis of bile duct lesion in PBC by the accentuated inflammatory microenvironment through recruiting monocytes and other inflammatory cells via SASP (Figure 5). SASPs in senescent BECs in PBC may contribute to activation of the innate immune system around injured bile ducts. Furthermore, it raises the possibility that once biliary senescence develops, the change in the tissue microenvironment wrought by the SASP may induce senescence of surrounding BECs another types of cells in appositive feedback loop (Figure 5).

The mechanisms that initiate and maintain SASPs have not been clarified, so far [33, 73–75]. It is plausible that these stresses may induce SASPs via a common mechanism in the senescent state, because various cellular stresses, such as oxidative stress and serum deprivation, induce SASPs in senescent BECs [32].

## 6. Summary

PBC is thought to result from a combination of multiple genetic factors and superimposed environmental triggers and apparently belongs to the “complex disease” category like most polygenic autoimmune diseases. Even though mitochondrial autoantigens and B-cell and T-cell autoepitopes have been well characterized in PBC, the pathogenesis of characteristic bile duct lesion and the exact role of AMA still remain to be elucidated. In this paper, we focused on a possible involvement of two novel cellular processes, autophagy and cellular senescence in BECs in bile duct lesions in PBC. Autophagy is expected to be a promising cellular mechanism involved in the autoimmune mechanism together with apoptosis. Cellular senescence may play a role in the immunopathology of BECs by expressing SASPs in PBC. Further studies are needed to disclose the autoimmune pathogenesis of PBC.
